# Cytoskeleton Force Exertion in Bulk Cytoplasm

**DOI:** 10.3389/fcell.2020.00069

**Published:** 2020-02-13

**Authors:** Jing Xie, Nicolas Minc

**Affiliations:** Institut Jacques Monod, Université de Paris, CNRS UMR 7592, Paris, France

**Keywords:** forces, microtubules, actin, cytoplasm, cytoskeleton

## Abstract

The microtubule and actin cytoskeletons generate forces essential to position centrosomes, nuclei, and spindles for division plane specification. While the largest body of work has documented force exertion at, or close to the cell surface, mounting evidence suggests that cytoskeletal polymers can also produce significant forces directly from within the cytoplasm. Molecular motors such as kinesin or dynein may for instance displace cargos and endomembranes in the viscous cytoplasm yielding friction forces that pull or push microtubules. Similarly, the dynamics of bulk actin assembly/disassembly or myosin-dependent contractions produce cytoplasmic forces which influence the spatial organization of cells in a variety of processes. We here review the molecular and physical mechanisms supporting bulk cytoplasmic force generation by the cytoskeleton, their limits and relevance to organelle positioning, with a particular focus on cell division.

## Introduction

The actin and microtubule (MT) cytoskeletons are universal force-generating machineries. They can deform and move organelles, cells and tissues on length scales spanning 3–4 orders of magnitude. Accordingly, the forces they produce vary in amplitude, from few pN for individual polymers and motors, up to tens of nN for larger assemblies (Howard, 2001). A large part of our current understanding of force generation comes from *in vitro* studies using purified filaments and motors. Those served to delineate the minimal organizational principles supporting force generation, and compute typical magnitudes. In general, one could segregate force generation into two simple classes: protrusive (pushing) and contractile (pulling) forces (Tolić-Nørrelykke, 2008; Svitkina, 2018). For example, MT and actin filaments typically produce pushing forces in the sub-pN range, when polymerizing against a barrier (Dogterom and Yurke, 1997; Footer et al., 2007). Conversely, filament shortening by depolymerization (Grishchuk et al., 2005; Jegou et al., 2013), or their association with molecular motors, like dynein for MTs (Mallik et al., 2004; Gennerich et al., 2007) and myosin for actin (Finer et al., 1994) entail significant modes of pulling force exertion. Integration of these force-generation units into larger networks with defined polarities, turn-over and organization allows to scale up force amplitude (Parekh et al., 2005; Laan et al., 2008; Thoresen et al., 2011; Laan et al., 2012; Bieling et al., 2016), as well as generate a plethora of remarkable network shape changes and movements, including contraction (Reymann et al., 2012; Foster et al., 2015), growth (Loisel et al., 1999; Ishihara et al., 2014), translation (Holy et al., 1997; Telley et al., 2012), rotation (Schaller et al., 2010; Sanchez et al., 2012), or oscillations (Placais et al., 2009), akin to *in vivo* situations.

Model systems, on the other hand, have defined the role and molecular regulation of cytoskeletal force generation *in vivo*, for key processes like spindle positioning, cell migration or tissue morphogenesis (Cramer et al., 1994; Gardel et al., 2010; McNally, 2013; Munjal and Lecuit, 2014; Svitkina, 2018). Cell division, for instance, which is the particular focus of this review, relies on a thorough spatio-temporal regulation of force-generation by both actin and MTs. In late prophase/metaphase, the mitotic spindle needs to be accurately positioned and oriented to specify the division plane (Morin and Bellaiche, 2011; Minc and Piel, 2012). In many cells, this process is orchestrated by astral MTs that push and/or pull onto spindle poles, to move and rotate the spindle into place (McNally, 2013). Spindle assembly and chromosome segregation are also heavily dependent on MT-based forces (Dumont and Mitchison, 2009). These forces contribute to shape and elongate the spindle, as well as move and position chromosomes in the metaphase plate and pull them apart later in anaphase. For cytokinesis, signals emanating from the spindle drive the assembly of a cytokinetic acto-myosin ring, which will contract to deform the plasma membrane, leading to the physical separation of daughter cells (Green et al., 2012). Thus, in a typical time of few minutes, MTs, actin, and their associated motors, can create a remarkable multitude of forces varying in amplitude and directions, that coexist in a cytoplasm as small as few microns.

Our appreciation of the importance of force generation by the cytoskeleton for cell and tissue behavior, contrasts with our current knowledge of the directionality and magnitudes of those forces *in vivo*. For instance, to date, only few reports have provided direct force measurements within live cells. These include measurements of contractile stresses generated by acto-myosin rings (Rappaport, 1967), forces associated with chromosome segregation (Nicklas, 1983), intracellular vesicle transport (Hendricks et al., 2012; Rai et al., 2013) and MT aster or spindle positioning (Garzon-Coral et al., 2016; Tanimoto et al., 2018). Whether cytoskeleton polymers apply pushing vs. pulling forces, to move or position nuclei or spindles, is also still surprisingly a matter of intense debate in many model systems (Grill et al., 2001; Bezanilla and Wadsworth, 2009; Garzon-Coral et al., 2016).

Another important conceptual notion in cytoskeleton force exertion is to distinguish between the role of forces applied from the cell surface vs. those applied directly from bulk cytoplasm. Because biological research has made a powerful use of model systems like yeast and adherent animal cells, which are small cells dominated by surface effects, the largest body of the literature has so far covered cytoskeletal force exertion at, or close to the cell surface. Examples range from actin polymerization for lamellipodium protrusion, contractile forces that regulate the actin cortex, or forces on astral MTs pulled by dynein anchored to the cortex during spindle positioning. Similarly, the proximity of glass coverslips in most *in vitro* assays, has limited our appreciation of the role of viscous or viscoelastic interactions of cytoskeleton filaments and motors with elements in bulk. Force exertion from bulk cytoplasm, may represent another important class of cytoskeletal regulation, which remains poorly appreciated and documented. We will review evidences supporting force exertion in bulk for both MTs and actin, discuss molecular and physical mechanisms, limits and relevance to organelle positioning and cell division.

## Microtubule Forces in Bulk Cytoplasm

### Experimental Evidences Supporting the Existence of MT Forces in the Cytoplasm

MTs are long and rigid polymers which grow from within the cytoplasm. In animal cells, MTs are dominantly nucleated from the centrosome, and radiate to form star-shape structures, called MT asters (Bornens, 2012). The net forces and torques exerted by astral MTs are instrumental to move, position, and orient centrosomes and associated nuclei and spindles (Morin and Bellaiche, 2011; Minc and Piel, 2012; McNally, 2013). Many seminal studies have clearly demonstrated that MTs can generate significant pushing forces *in vivo* to promote nucleus or spindle centration (Tran et al., 2001; Tolić-Nørrelykke et al., 2004). Astral MTs interaction with cortical dynein is also widely accepted to create pulling forces that position and orient spindles in a multitude of cell types (Grill et al., 2001; Gönczy, 2008). Although such MT cortical forces are typically considered as dominant modes of force generation (Tolić-Nørrelykke, 2008), experiments based on the local manipulation of MTs in asters, support that MTs can also pull directly from the cytoplasm without contacting the cell surface (Reinsch and Gönczy, 1998; Wühr et al., 2009; Mitchison et al., 2012).

**FIGURE 1:**
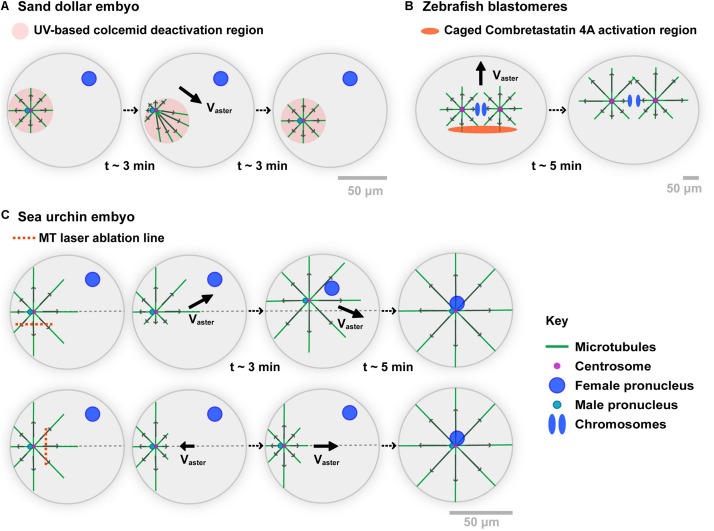
Experimental evidences of the role of MT cytoplasmic pulling forces for aster motion. **(A)** A Sand dollar embryo is fertilized in the presence of the MT drug Colcemid. Using UV light, MTs are allowed to polymerize in specific regions. When moving the UV region, the sperm aster migrates to the center of the new region, following the direction of longer MTs. As MTs do not contact any cortex, those experiments support that forces exerted by MTs in bulk cytoplasm can drive aster motion and positioning. Modified from Hamaguchi and Hiramoto (1986). **(B)** Zebrafish 2-cell stage blastomeres are incubated in caged combretastatin 4A. Local UV-based uncaging allows for locally depolymerizing MTs in asters. Asters move following their longer MTs, long before those contact the cortex, supporting MT cytoplasmic force exertion. Modified from Wuhr et al. (2010). **(C)** Sea urchin embryos are fertilized, and MT centering asters are cut with a UV-laser along the indicated lines while they move to the cell center. Laser ablation on the side of asters causes a transient deviation toward the top of the cell, supporting MT bulk pulling. MT regrowth promote a return of the aster to the centration path. Similarly, laser cuts at aster front cause aster to transiently step back, and move again to the cell center upon MT regrowth. Modified from Tanimoto et al. (2016).

The seminal Colcemid-UV experiments by Hamaguchi and Hiramoto in the 80s, constitute the first important support for MT force exertion in bulk (Hamaguchi and Hiramoto, 1986) (Figure 1A). Using the centration of sperm asters in large Sand dollar (echinoderm) embryos, they depolymerized all MTs with Colcemid, a powerful MT inhibitor which can be inactivated with UV light. Local UV-based inactivation allows MTs to regrow in defined sub-cellular regions. Importantly, as long as the UV light is on, those regions remain stable against diffusion of inactivated molecules, given the large excess of Colcemid in the medium. Thus, this assay allows to create asymmetric asters with MTs differing in length. Remarkably, asters moved to the center of UV zones, following the direction of longer MTs, much like they normally do when migrating to the center of the whole cell (Chambers, 1939). Importantly, multiple modulations of this Colcemid-UV assay clearly ruled out any requirements for a contact between MTs and the cortex, demonstrating that MT forces exerted in bulk can displace asters and centrosomes (Hamaguchi and Hiramoto, 1986).

Recent experiments in large zebrafish blastomeres, used an inverse approach based on an engineered caged version of the MT drug Combretastatin 4A (Wuhr et al., 2010; Figure 1B). Local and transient UV-based uncaging of the drug allows for depolymerizing a fraction of MTs, creating asymmetric asters that again moved toward the sides of longer MTs. Net motion occurred long before MTs reached the cortex, supporting that MT forces exerted in bulk can displace large asters. A last version of those experiments, involved UV laser-based ablation of parts of centering sperm asters in sea urchin embryos (Tanimoto et al., 2016; Figure 1C). Cutting MTs along a line parallel to the aster centering path, yielded a systematic deviation away from the ablation line, suggesting that uncut MTs predominantly exert pulling forces. Soon after ablation, MTs regrowth and restored the position of the centrosome on the centered path. Similarly, cutting MTs at the aster front caused aster to transiently stop or even step back toward the cortex. Thus, at least in large eggs and blastomeres, those different assays provide strong evidence that MT cytoplasmic forces are dominant in driving the motion of centrosomes, nuclei, and asters.

### Mechanisms Supporting Microtubule Force Generation in the Cytoplasm

**FIGURE 2:**
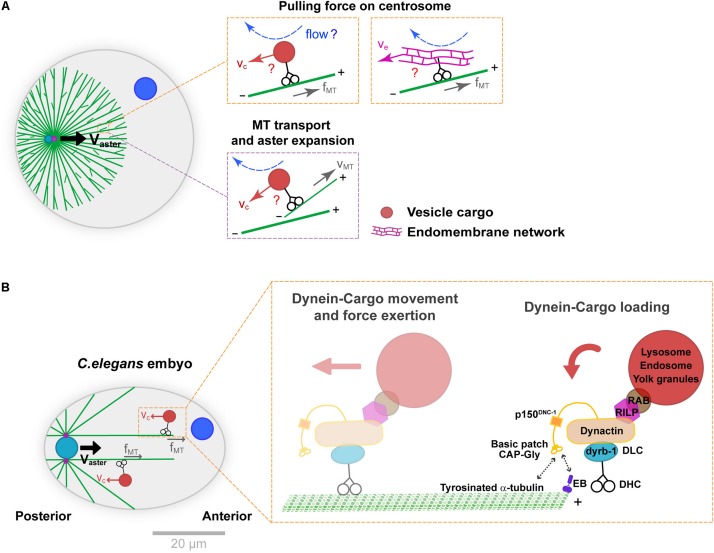
Mechanisms supporting dynein cytoplasmic pulling force exertion on MTs. **(A)** Aster centration by dynein-mediated forces in bulk. Dynein may transport vesicle-cargos (e.g., endosomes or lysosomes) or large endomembrane networks like the endoplasmic reticulum (orange insets). The motion of those cargos in the viscous cytoplasm creates flows and drag forces that pull on MTs and ultimately centrosomes. This module coupled to front-rear asymmetries in MT length in asters generates a net centering force. Similar cytoplasmic forces may also transport MTs to the periphery of the asters, contributing to aster expansion (purple insets). **(B)** Specific subunits of the dynein-dynactin complex and adaptors thought to mediate dynein-cargo loading as well as cargo motion, during *C. elegans* pro-nuclear centration. The cytoplasmic dynein-cargo is loaded onto MTs by interacting with EB proteins at the plus end or tyrosinated α-tubulin along the MTs. Modified according to Kimura and Kimura (2011) and Barbosa et al. (2017). Scale bar, 20 μm.

In the systems mentioned above, as well as in many other cell types, the main motor driving centrosome motion is dynein (Gönczy et al., 1999; O’Connell and Wang, 2000; Wuhr et al., 2010; Tanimoto et al., 2016; Cheng and Ferrell, 2019). Dynein anchoring and activation at the cortex have been extensively documented, but how this minus-end directed motor may load onto MTs and pull them in bulk is less well understood. One possibility is that dynein could be anchored on relatively static structures in bulk, like a stiff actin or intermediate filament network (Reinsch and Gönczy, 1998). Although actin appears to be dispensable for MT bulk force exertion in some systems (Tanimoto et al., 2016; Cheng and Ferrell, 2019), the role of intermediate filaments has not been tested thoroughly to date. An alternative scenario, introduced in another classical paper by Hamagushi and Hiramoto is that small objects like vesicle cargos moving along MTs, could interact hydrodynamically with the viscous cytoplasm, and yield frictional forces that pull onto MTs (Hamaguchi et al., 1986; Figure 2A). This hypothesis was based on the observation that some particles like polystyrene beads injected in eggs, may exhibit highly persistent minus-end motions along astral MTs. Because vesicle-cargos like endosomes, lysosomes, mitochondria or yolk granules, as well as larger endomembranes of the endoplasmic reticulum, are typically transported by dynein along MTs, they could provide natural viscous anchors for MTs to pull in bulk (Terasaki and Jaffe, 1991; Reinsch and Gönczy, 1998; Figure 2A).

This model received the best support from genetic functional analyses in the model *C. elegans* one-cell embryo (Figure 2B). Upon fertilization, much like in echinoderms mentioned above and most animal eggs, the male pro-nucleus moves to the cell center guided by centering sperm asters. Building on large-scale protein knock-down analyses, Kimura and colleagues defined the role of the dynein light chain subunit dyrb-1, as well as adaptors RILP and Rab7 that connect dynein to vesicle cargos for centrosome centration (Kimura and Kimura, 2011). RNAi of those factors significantly reduced endosomes and lysosomes minus-end directed motion and net aster centration speed, bringing key molecular insights into how organelle motion could mediate dynein force exertion from the cytoplasm. Recent studies of the dynein-dynactin complex in the same system, revealed mechanisms controlling the loading of vesicle cargo-dynein complexes onto MTs. These showed interactions between the dynactin subunit p150 GAP-Gly domain with MT +TIP EB proteins, as well as with tyrosinated α-tubulin. Specific mutants of this domain yielded concomitant defects in endosome motion and aster centration, giving further support to the vesicle-based pulling model in *C. elegans* (Barbosa et al., 2017). Because organelle trafficking along MTs is a feature of all eukaryotic cells, this mechanism could potentially influence nuclear or spindle positioning in many cells, not only zygotes. However, it remains unclear whether the prominent –end directed motion of endosomes and lysosomes reported in *C. elegans* represents a conserved feature, or if competing activities with +end directed kinesins could cancel out this effect in other cell types.

### Implications of MT Cytoplasmic Forces for Aster Positioning

How might dynein pulling in bulk cytoplasm influence the stereotypical centering motion of asters? The most-accepted design is that longer MTs should accumulate more dyneins and cargos, allowing them to exert more pulling forces on centrosomes (O’Connell and Wang, 2000; Vallee and Stehman, 2005; Wuhr et al., 2010; Minc et al., 2011). This design provides a robust mean to convert aster shape into a net force, and account for aster centration, as well as all above-mentioned experiments where MTs are locally depleted (Tanimoto et al., 2016; Haupt and Minc, 2018). This design received important support from multiple theoretical modeling work (Kimura and Onami, 2005; Hendricks et al., 2012; Letort et al., 2016; Tanimoto et al., 2016). Hydrodynamic simulations even allowed to account for the resultant flows and forces in the fluid associated with length-dependent MT pulling in bulk (Shinar et al., 2011; Nazockdast et al., 2017). Yet, to date, there is no experimental or theoretical validation that vesicle transport will be an efficient mean for force exertion on MTs, nor that vesicles actually accumulate onto MTs in a length-dependent manner.

**FIGURE 3:**
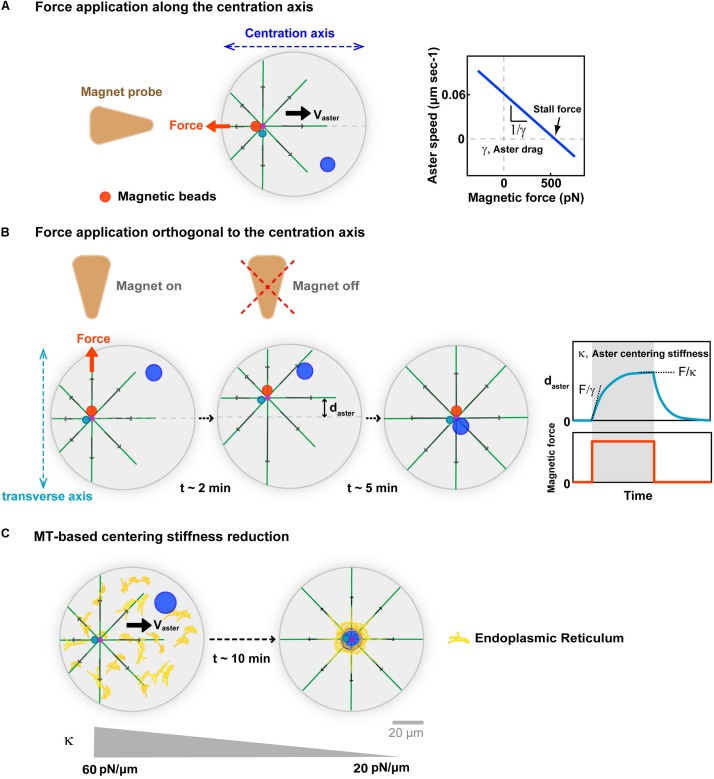
Implications of MT cytoplasmic forces for aster centration. **(A)** External calibrated magnetic forces applied with magnetic tweezers on the aster center, through minus-end accumulated beads in sea urchin embryos. Forces applied along the centering axis serve to derive force-velocity relationships for centering asters. This relationships is mostly linear supporting a motion limited by viscous interactions, with the slope allowing to compute an effective drag. The intersection of the line with the x-axis yields estimates of the aster endogenous force of ∼600 pN. Modified according to Tanimoto et al. (2018). **(B)** Forces applied orthogonal to the centering axis serve to characterize MT-based visco-elastic forces stabilizing the centered position of the aster in the transverse axis during centration. Blue curve: Typical experimental displacement-time curve, allowing to compute visco-elastic parameters of MT-based centering system. Upon force application, the aster moves toward the magnet until reaching an equilibrium plateau, and recoils back to the center after releasing the force, behaving as a spring. Computation of the centering stiffness and the drag from the plateau and the slope of the curve. Modified from Tanimoto et al. (2018). **(C)** Successive force measurements showed that aster centering stiffness becomes reduced during aster centration (Salle et al., 2019). Proposed model for how cytoplasmic forces may become less efficient during centration, and yield to this stiffness reduction: As the aster centers, dynein drags endomembranes to the cell center, depleting the rest of the cytoplasm, and thereby rendering cytoplasm force exertion less efficient. Modified from Terasaki and Jaffe (1991) and Salle et al. (2019).

One important mean to connect the length-scales of single force-generation events to that of whole asters, is to compute the net forces associated with centering asters *in vivo* (Wu et al., 2016; De Simone et al., 2018). Building on injected magnetic beads which exhibit persistent minus-end directed motion and stick to centrosomes, we recently used *in vivo* magnetic tweezers, to derive force-velocity relationships for centering sperm asters in sea urchin eggs (Tanimoto et al., 2018; Figure 3A). These allowed to extract a stall force that equilibrates the endogenous aster net centering force of ∼600 pN. This force is equivalent to a net excess of hundreds of motors, or tens of vesicle cargos at the aster front (Rai et al., 2013). While this may seem realistic, the lack of understanding on how much of the vesicle force is actually transmitted to single MTs or to the centrosome, precludes any firm conclusion for the exact mechanism that generates such large forces. Asters are bushy, with MTs often unconnected to the rest of the network, and whether vesicle motion will just transport MTs to the periphery of the aster, promoting aster expansion, drive network rearrangements, or actually pull on the centrosome is not clear (Mitchison et al., 2012; Ishihara et al., 2014; Figure 2A). In addition, the motion of vesicle cargos in dense asters, is a sophisticated hydrodynamic problem, resembling that of a sphere moving in a closed pipe, which could significantly limit dynein force transmission to MTs (Happel and Brenner, 2012; Nazockdast et al., 2017).

*In vivo* magnetic forces can also serve to move the centrosome away from the cell center, and assay their ability to re-find the cell center. Such experiments were first performed on centered mitotic spindles in *C. elegans* zygotes (Garzon-Coral et al., 2016). They revealed the existence of MT-dependent visco-elastic forces, which maintain the spindle in the cell center that were proposed to emerge from astral MT pushing forces on the cortex. During aster centration in sea urchin embryo, applying magnetic forces orthogonal to the centering axis, also yielded similar MT visco-elastic forces, which maintain the centrosome along the centering path (Figure 3B). The centering stiffness provided by MTs is ∼60 pN/μm. Thus, a displacement 1 μm away from the centering path, creates a net imbalance of 60 pN in MT forces from the two sides on the centrosome. We interpreted this imbalance as a natural consequence of the length-dependent MT pulling model: as the aster is moved away from the center MTs become longer in the other direction, exerting a net restoring force proportional to the net displacement of the aster center (Tanimoto et al., 2018; Dmitrieff and Minc, 2019). Remarkably, this MT-based centering stiffness becomes gradually smaller as the sperm aster approaches the cell center (Salle et al., 2019). This reduction could also be a mere consequence of the cytoplasmic pulling model. As vesicles and/or endomembranes are transported to the aster center by dynein, they become gradually depleted from the rest of the cytoplasm, thereby reducing the amount of viscous anchors for dynein to pull on MTs (Figure 3C). Accordingly, the endoplasmic reticulum exhibits a massive accumulation around centering sperm asters (Terasaki and Jaffe, 1991). Whether other cargos exhibit similar accumulation remains to be determined, and could represent a qualitative criteria to identify the specific dynein cargos supporting bulk pulling.

Finally, in addition to centering asters and promoting symmetric division, length-dependent bulk MT forces may also influence division orientation. During early embryonic cell cycles, asters duplicate and orient to specify the geometry of early division patterns. One remarkable properties of those divisions, is that they usually orient along the long axis of the cell. This process, which is thought to be a general property of many cells, was first reported in the nineteenth century in Oskar Hertwig’s classical rules (Hertwig, 1884; Minc and Piel, 2012). Length-dependent MT forces allow to orient aster pairs along the long axis of cells, thereby explaining those century-old division rules (Wuhr et al., 2010; Minc et al., 2011; Pierre et al., 2016; Haupt and Minc, 2018). In different embryos, aster geometries may be influenced not only by cell shape, but also by the presence of neighboring asters, or yolk layers which affect MT growth. Combinations of those factors ultimately dictate aster shapes and consequent position and orientation, accounting for a large part of the diversity of cleavage patterns seen in different embryos (Pierre et al., 2016). Thus, MT length-dependent bulk pulling forces constitutes a generic design which may account for essential self-organizing properties of asters and early embryos.

## Actin-Based Forces in Bulk Cytoplasm

### The Actin Comet-Tail Model

Actin is also responsible for the generation of large forces, in essential instances like cell migration, cytokinesis, and shape changes (Gardel et al., 2010; Green et al., 2012; Munjal and Lecuit, 2014; Svitkina, 2018). The largest part of F-actin in animal cells resides in the cell cortex, underneath the plasma membrane. As a consequence, addressing the role of bulk F-actin has long remained difficult, in part because the visualization of filaments deep in the cytoplasm is often obscured by the very bright cortex (Field and Lenart, 2011). The development of actin live probes including life-act and Utrophin has allowed important new discoveries on the specific function of bulk actin (Li et al., 2008; Field et al., 2011; Almonacid et al., 2015; Shamipour et al., 2019).

**FIGURE 4:**
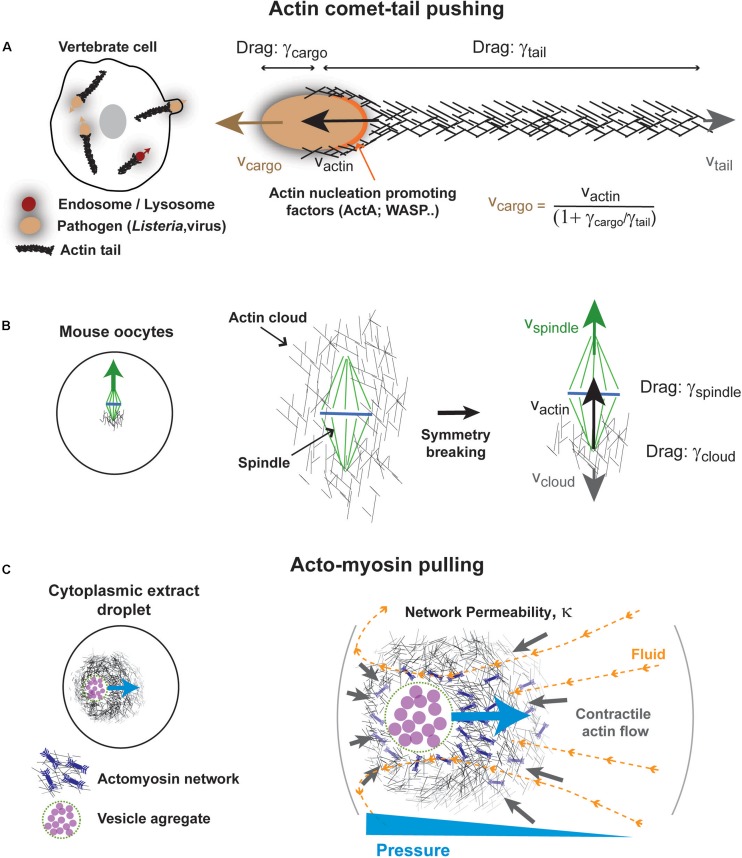
Some mechanisms for actin-based force exertion in bulk. **(A)** Actin comet tail model for actin based cytoplasmic pushing. Comet tails grow at the surface of different microbes, such as *Listeria monocytogenes*, or even at the surface of intracellular vesicles. Actin polymerization promoted by local actin regulators such as ActA for *Listeria* and Wasp for lysosomes promotes the growth of a branched actin network, by activating host arp2/3 complex. Assuming pure viscous interactions of both cargos and tails with the cytoplasm, the propulsive speed of the cargo is linked to that of actin growth speed by their drag ratio as indicated. **(B)** Meiotic spindle asymmetric displacement in mouse oocytes. An actin cloud forms around spindle and chromosomes as a result of formin-nucleation activity around spindles or chromosomes. This cloud breaks symmetry and pushes the spindle upward invoking similar physical principles as for comet-tails. Modified from Li et al. (2008). **(C)** Self-organized acto-myosin contractile networks in extracts encapsulated in droplets can drive actin and fluid flows. Darcy flows through the permeable network create a pressure gradient that pushes the network toward the cell center. Modified from Ierushalmi et al. (2019).

One well documented class of mechanism for force exertion by F-actin in bulk cytoplasm, comes from studies of the propulsion of the pathogenic bacterium *Listeria monocytogenes* (Figure 4A). Upon infection, this microbe hijacks the actin cytoskeleton machinery of the host cell to grow a long “comet tail” made of highly reticulated actin filaments that propel the bacterium in the cytoplasm (Tilney and Portnoy, 1989; Theriot et al., 1992; Tilney et al., 1992). At the surface of bacterial poles, a nucleation promoting factor called ActA recruits and activates host Arp2/3 complex that promotes branched actin growth (Welch et al., 1997). Multiple seminal studies reconstituted actin comets at the surface of beads, vesicles, droplets, in cytoplasmic extracts, or in minimal solutions (Theriot et al., 1992; Cameron et al., 1999; Loisel et al., 1999; Pantaloni et al., 2001; Bernheim-Groswasser et al., 2002; Giardini et al., 2003; Trichet et al., 2007; Delatour et al., 2008). Those served to draw a large part of our current understanding of actin assembly and force generation, needed for other processes like lamellipodium based migration (Theriot et al., 1994; Laurent et al., 1999; Pantaloni et al., 2001). In addition, comet tails also form around other pathogens including viruses (Welch and Way, 2013), as well as at the surface of endosomes, lysosomes or yolk granules (Taunton et al., 2000; Shamipour et al., 2019), suggesting that they could promote the intracellular motion of many types of cargos in the cytoplasm.

The forces developed by actin comet-tails can be enormous, ranging from hundreds of pN to few nN (Giardini et al., 2003; McGrath et al., 2003; Upadhyaya et al., 2003; Marcy et al., 2004; Plastino and Sykes, 2005). In addition to propelling the bacterium, those forces may help the microbe to eventually deform and breach the host membrane for propagation to neighboring cells (Portnoy et al., 2002; Pizarro-Cerda and Cossart, 2006). The propulsive speeds of *Listeria* are also impressive, reaching up to ∼1 μm/s, and are nearly equal to that of actin tail growth speeds (Dabiri et al., 1990). In the absence of external forces, the ensemble cargo (e.g., bacteria, beads…) plus actin tail is an isolated system (Prost et al., 2008). Consequently, which of the cargo or the tail will move in the laboratory frame is dictated by the ratio of the viscous drags of each object. Those drags depend on their respective geometries and the viscosity of the cytoplasm. Given the large density of filaments in the actin tail, its drag may be well approximated with that of a solid cylinder, which increases linearly with the tail length (Happel and Brenner, 2012). Thus, if the tail is much shorter than the cargo radius, it will elongate with no net displacement of the cargo. Once the tail reaches a similar length than the cargo, they will move in opposite directions at similar speeds. However, when the length of the tail exceeds the radius of the cargo, the drag of the tail will dominate and propel the cargo at a speed that is linked to that of actin tail growth, by: $V_{c\,a\,r\,g\,o}=\frac{V_{a\,c\,t\,i\,n}}{\left(1+\gamma_{c\,a\,r\,g\,o}/\gamma_{t\,a\,i\,l}\right)}$ (Prost et al., 2008). *In vivo*, actin tails are typically 20–30X longer than their cargos, and thus the speed of the cargos, can be very well approximated with that of actin growth (Theriot et al., 1992). Thus, as for MTs and vesicles moved by dynein discussed above, the comet tail-based motion exemplifies how a pure viscous interaction with bulk cytoplasm, can support force exertion and rapid net motion of cargos.

However, the bacterium or other cargos are often subjected to external forces, when encountering other organelles or cellular networks, or when pushing against the membrane. For instance, *Listeria’s* speed depends on its location within the host cells, which has been used to argue for an influence of the local density of cytoskeletal networks of actin, MTs or intermediate filaments (Giardini and Theriot, 2001; Giardini et al., 2003). During microbe escape, actin tails also appear to connect to the cortex of evaginating membranes, raising the possibility that the tail could push on more elastic or viscoelastic elements to support forward progression of the bacterium against load (Sechi et al., 1997). Because some actin tails appear smaller than their cargos, it is likely that visco-elastic interactions of the tail with other elements may be required in some instances (Shamipour et al., 2019).

In the context of cell division, the comet-tail pushing mechanism has been used to account for the decentration of the meiotic spindle in mouse oocytes (Figure 4B). Meiotic spindle displacement from the cell center to the nearest cortex, has been the subject of significant debates, arguing for the prominence of contractile forces from an acto-myosin network connected to the cortex vs. pushing from an actin cloud (Azoury et al., 2008; Li et al., 2008; Schuh and Ellenberg, 2008; Bezanilla and Wadsworth, 2009). The pushing mechanism is based on the observation of a large formin-nucleated F-actin cloud surrounding spindles and chromosomes, which breaks symmetry to become asymmetric at the onset of spindle displacement (Li et al., 2008). Notably, the cloud lagging the spindle has a size that roughly equals or is smaller than the spindle. Thus, following above arguments, whether the cloud solely uses viscous interaction with the cytoplasm to move spindles and chromosomes, or if it pushes on other bulk visco-elastic networks remains an interesting open question.

### Actin-Based Pulling in Bulk

Acto-myosin based contraction is the main force that drives cell shape changes, cytokinesis, and many tissue rearrangements (Green et al., 2012; Munjal and Lecuit, 2014; Chugh and Paluch, 2018). Acto-myosin networks are most prominent at the cortex, and their local contraction can yield actin flows that concentrate actin and myosin toward the site of highest contractility (Callan-Jones and Voituriez, 2016). Provided balanced disassembly at this site, the network can exhibit steady state gradients in actin density and flows, which are thought to contribute to cell polarization, as well as multiple aspects of cell migration (Mayer et al., 2010; Liu et al., 2015; Maiuri et al., 2015; Ruprecht et al., 2015). Can similar mechanisms organize the bulk cytoplasm in such events as nuclear or spindle positioning? Bulk cytoplasmic extracts can exhibit massive acto-myosin dependent contractility (Field and Lenart, 2011; Field et al., 2011), suggesting that those types of forces exist in the cytoplasm and could contribute to organize the cell interior.

Recent *in vitro* studies provide important support for the relevance of bulk actin flows to cellular spatial organization (Figure 4C). These are based on the encapsulation of crude frog extracts supplemented with energy mix in water-in-oil cell-like droplets (Pinot et al., 2012; Tang et al., 2018; Malik-Garbi et al., 2019). Remarkably, those extracts rapidly self-organize to create sustained gradients of acto-myosin contractility with actin flowing from the droplet surface to its center (Pinot et al., 2012; Malik-Garbi et al., 2019). Flow properties depend on factors which control actin assembly and disassembly, as well as network connectivity (Malik-Garbi et al., 2019).

Remarkably, this network self-organizes around an aggregate of vesicles, which are dragged by actin flows, and locates to the precise droplet center. When this vesicle aggregate is moved away from the droplet center with magnetic tweezers, acto-myosin flows exert a net centering force, which moves the aggregate back to the droplet center. Thus, much like MT asters, disordered acto-myosin networks can act as elastic springs related to cell geometry. Their restoring elastic forces also emerge from a shape anisotropy of the network. When the network center is close to the cortex, the network grows larger away from the cortex, and drags more fluid, thereby creating a pressure gradient that pushes it back to the droplet center. This pressure gradient depends on the permeability of the network as well as fluid flow speeds and organization. Thus, this mechanism may have similarities with MT aster centering from bulk dynein forces. Whether this mechanism directly contributes to center organelles like the nucleus in cells remains to be tested, but similar bulk actin flows, have been shown to exist in fish embryos and influence the organization of their cytoplasm (Shamipour et al., 2019).

## Discussion

Several decades after the pioneering experiments by Hamaguchi and Hiramoto (1986), our appreciation of cytoskeleton force exertion in bulk remains narrow. This in part because those effects are best studied in large cells, such as eggs and blastomeres, which are less studied than yeast or adherent vertebrate cells. However, vesicles trafficking along MTs or actin growth in bulk are highly conserved processes, and thus it is likely that such forces exist even in small asters and cells (Telley et al., 2012; Wu et al., 2012). Whether their impact remains negligible as compared to surface forces is an exciting question for the future of the field. The physical mechanisms supporting force exertion from the cytoplasm are also still in their infancy. For instance, it is not clear if the radial organization in the standard model is actually required for aster motion based on cytoplasmic forces. Recent data with *in vitro* frog extracts actually suggest that asters can self-organize, center nuclei and organize cleavage patterns, even in the absence of centrosomes (Cheng and Ferrell, 2019). Akin to the situation depicted in Figure 4C for bulk acto-myosin networks, a disordered MT network with a rough polarity and front-rear asymmetry, could in principle propel itself to the cell center by moving fluid through it using dynein-powered organelle/endomembrane transport. The efficiency of such self-propulsion would depend on parameters such as network permeability and density of viscous pullers, which are parameters difficult to map directly *in vivo* (Nazockdast et al., 2017; Tanimoto et al., 2018; Ierushalmi et al., 2019). Finally, whether cytoplasm viscosity is sufficient for significant force exertion, or if cytoskeleton polymers take support on more elastic or viscoelastic elements is not known. Although actin is not required for aster motion based on cytoplasmic pulling (Kimura and Kimura, 2011; Minc et al., 2011; Tanimoto et al., 2016), it may potentially influence their motion properties, by modulating the viscosity of the cytoplasm. Future work integrating the physical properties of the cytoplasm with cytoskeletal forces, to understand the motion of large objects like asters, spindles or nuclei shall strongly impact the field of cell division.

## Author Contributions

JX and NM designed the content, made figures, and wrote text.

## Conflict of Interest

The authors declare that the research was conducted in the absence of any commercial or financial relationships that could be construed as a potential conflict of interest.

## References

[B1] Almonacid M. Ahmed W. W. Bussonnier M. Mailly P. Betz T. Voituriez R. (2015). Active diffusion positions the nucleus in mouse oocytes. *Nat. Cell Biol.* 17 470–479. 10.1038/ncb3131 25774831

[B2] Azoury J. Lee K. W. Georget V. Rassinier P. Leader B. Verlhac M. H. (2008). Spindle positioning in mouse oocytes relies on a dynamic meshwork of actin filaments. *Curr. Biol.* 18 1514–1519. 10.1016/j.cub.2008.08.044 18848445

[B3] Barbosa D. J. Duro J. Prevo B. Cheerambathur D. K. Carvalho A. X. Gassmann R. (2017). Dynactin binding to tyrosinated microtubules promotes centrosome centration in *C. elegans*. *PLoS Genet.* 13:e1006941. 10.1371/journal.pgen.1006941 28759579 PMC5552355

[B4] Bernheim-Groswasser A. Wiesner S. Golsteyn R. M. Carlier M. F. Sykes C. (2002). The dynamics of actin-based motility depend on surface parameters. *Nature* 417 308–311. 10.1038/417308a 12015607

[B5] Bezanilla M. Wadsworth P. (2009). Spindle positioning: actin mediates pushing and pulling. *Curr. Biol.* 19 R168–R169. 10.1016/j.cub.2008.12.026 19243693 PMC2848404

[B6] Bieling P. Li T. D. Weichsel J. Mcgorty R. Jreij P. Huang B. (2016). Force feedback controls motor activity and mechanical properties of self-assembling branched actin networks. *Cell* 164 115–127. 10.1016/j.cell.2015.11.057 26771487 PMC5033619

[B7] Bornens M. (2012). The centrosome in cells and organisms. *Science* 335 422–426. 10.1126/science.1209037 22282802

[B8] Callan-Jones A. C. Voituriez R. (2016). Actin flows in cell migration: from locomotion and polarity to trajectories. *Curr. Opin. Cell Biol.* 38 12–17. 10.1016/j.ceb.2016.01.003 26827283

[B9] Cameron L. A. Footer M. J. Van Oudenaarden A. Theriot J. A. (1999). Motility of ActA protein-coated microspheres driven by actin polymerization. *Proc. Nat. Acad. Sci. U.S.A.* 96 4908–4913. 10.1073/pnas.96.9.4908 10220392 PMC21790

[B10] Chambers E. L. (1939). The movement of the egg nucleus in relation to the sperm aster in the echinoderm egg. *J. Exp. Biol.* 16 409–424.

[B11] Cheng X. Ferrell J. E. (2019). Spontaneous emergence of cell-like organization in *Xenopus* egg extracts. *Science* 366 631–637. 10.1126/science.aav7793 31672897 PMC7839252

[B12] Chugh P. Paluch E. K. (2018). The actin cortex at a glance. *J. Cell Sci.* 131:jcs186254. 10.1242/jcs.186254 30026344 PMC6080608

[B13] Cramer L. P. Mitchison T. J. Theriot J. A. (1994). Actin-dependent motile forces and cell motility. *Curr. Opin. Cell Biol.* 6 82–86. 10.1016/0955-0674(94)90120-1 8167030

[B14] Dabiri G. A. Sanger J. M. Portnoy D. A. Southwick F. S. (1990). Listeria monocytogenes moves rapidly through the host-cell cytoplasm by inducing directional actin assembly. *Proc. Natl. Acad. Sci. U.S.A.* 87 6068–6072. 10.1073/pnas.87.16.6068 2117270 PMC54473

[B15] De Simone A. Spahr A. Busso C. Gönczy P. (2018). Uncovering the balance of forces driving microtubule aster migration in *C. elegans* zygotes. *Nat. Commun.* 9:938. 10.1038/s41467-018-03118-x 29507295 PMC5838244

[B16] Delatour V. Helfer E. Didry D. Le K. H. Gaucher J. F. Carlier M. F. (2008). Arp2/3 controls the motile behavior of N-WASP-functionalized GUVs and modulates N-WASP surface distribution by mediating transient links with actin filaments. *Biophys. J.* 94 4890–4905. 10.1529/biophysj.107.118653 18326652 PMC2397363

[B17] Dmitrieff S. Minc N. (2019). Scaling properties of centering forces. *Europhys. Lett.* 125:48001 10.1209/0295-5075/125/48001

[B18] Dogterom M. Yurke B. (1997). Measurement of the force-velocity relation for growing microtubules. *Science* 278 856–860. 10.1126/science.278.5339.856 9346483

[B19] Dumont S. Mitchison T. J. (2009). Force and length in the mitotic spindle. *Curr. Biol.* 19 R749–R761. 10.1016/j.cub.2009.07.028 19906577 PMC2791830

[B20] Field C. M. Lenart P. (2011). Bulk cytoplasmic actin and its functions in meiosis and mitosis. *Curr. Biol.* 21 R825–R830. 10.1016/j.cub.2011.07.043 21996509

[B21] Field C. M. Wühr M. Anderson G. A. Kueh H. Y. Strickland D. Mitchison T. J. (2011). Actin behavior in bulk cytoplasm is cell cycle regulated in early vertebrate embryos. *J. Cell Sci.* 124 2086–2095. 10.1242/jcs.082263 21610091 PMC3104037

[B22] Finer J. T. Simmons R. M. Spudich J. A. (1994). Single myosin molecule mechanics: piconewton forces and nanometre steps. *Nature* 368 113–119. 10.1038/368113a0 8139653

[B23] Footer M. J. Kerssemakers J. W. Theriot J. A. Dogterom M. (2007). Direct measurement of force generation by actin filament polymerization using an optical trap. *Proc. Natl. Acad. Sci. U.S.A.* 104 2181–2186. 10.1073/pnas.0607052104 17277076 PMC1892916

[B24] Foster P. J. Furthauer S. Shelley M. J. Needleman D. J. (2015). Active contraction of microtubule networks. *elife* 4:e10837. 10.7554/eLife.10837 26701905 PMC4764591

[B25] Gardel M. L. Schneider I. C. Schaus Y. A. Waterman C. M. (2010). Mechanical integration of actin and adhesion dynamics in cell migration. *Ann. Rev. Cell Dev. Biol.* 26 315–333. 10.1146/annurev.cellbio.011209.122036 19575647 PMC4437624

[B26] Garzon-Coral C. Fantana H. A. Howard J. (2016). A force-generating machinery maintains the spindle at the cell center during mitosis. *Science* 352 1124–1127. 10.1126/science.aad9745 27230381 PMC6535051

[B27] Gennerich A. Carter A. P. Reck-Peterson S. L. Vale R. D. (2007). Force-induced bidirectional stepping of cytoplasmic dynein. *Cell* 131 952–965. 10.1016/j.cell.2007.10.016 18045537 PMC2851641

[B28] Giardini P. A. Fletcher D. A. Theriot J. A. (2003). Compression forces generated by actin comet tails on lipid vesicles. *Proc. Natl. Acad. Sci. U.S.A.* 100 6493–6498. 10.1073/pnas.1031670100 12738883 PMC164474

[B29] Giardini P. A. Theriot J. A. (2001). Effects of intermediate filaments on actin-based motility of *Listeria monocytogenes*. *Biophys. J.* 81 3193–3203. 10.1016/s0006-3495(01)75955-3 11720985 PMC1301779

[B30] Gönczy P. (2008). Mechanisms of asymmetric cell division: flies and worms pave the way. *Nat. Rev. Mol. Cell Biol.* 9 355–366. 10.1038/nrm2388. 18431399

[B31] Gönczy P. Pichler S. Kirkham M. Hyman A. A. (1999). Cytoplasmic dynein is required for distinct aspects of MTOC positioning, including centrosome separation, in the one cell stage *Caenorhabditis elegans* embryo. *J. Cell Biol.* 147 135–150. 10.1083/jcb.147.1.135 10508861 PMC2164971

[B32] Green R. A. Paluch E. Oegema K. (2012). Cytokinesis in animal cells. *Ann. Rev. Cell Dev. Biol.* 28 29–58. 10.1146/annurev-cellbio-101011-155718 22804577

[B33] Grill S. W. Gönczy P. Stelzer E. H. Hyman A. A. (2001). Polarity controls forces governing asymmetric spindle positioning in the *Caenorhabditis elegans* embryo. *Nature* 409 630–633. 10.1038/35054572 11214323

[B34] Grishchuk E. L. Molodtsov M. I. Ataullakhanov F. I. Mcintosh J. R. (2005). Force production by disassembling microtubules. *Nature* 438 384–388. 10.1038/nature04132 16292315

[B35] Hamaguchi M. Hamaguchi Y. Hiramoto Y. (1986). Microinjected polystyrene beads move along astral rays in sand dollar eggs. *Dev. Growth Differ.* 28 461–470. 10.1111/j.1440-169x.1986.00461.x 37282053

[B36] Hamaguchi M. S. Hiramoto Y. (1986). Analysis of the role of astral rays in pronuclear migration in sand dollar eggs by the colcemid−UV method. *Dev. Growth Differ.* 28 143–156. 10.1111/j.1440-169x.1986.00143.x 37280905

[B37] Happel J. Brenner H. (2012). *Low Reynolds Number Hydrodynamics: With Special Applications to Particulate Media.* New York, NY: Springer Science & Business Media.

[B38] Haupt A. Minc N. (2018). How cells sense their own shape - mechanisms to probe cell geometry and their implications in cellular organization and function. *J. Cell Sci.* 131:jcs214015. 10.1242/jcs.214015 29581183

[B39] Hendricks A. G. Holzbaur E. L. Goldman Y. E. (2012). Force measurements on cargoes in living cells reveal collective dynamics of microtubule motors. *Proc. Natl. Acad. Sci. U.S.A.* 109 18447–18452. 10.1073/pnas.1215462109 23091040 PMC3494964

[B40] Hertwig O. (1884). *Das Problem der Befruchtung und der Isotropie des Eies, Eine Theory der Vererbung.* Frankfurt: Fischer Publishing.

[B41] Holy T. E. Dogterom M. Yurke B. Leibler S. (1997). Assembly and positioning of microtubule asters in microfabricated chambers. *Proc. Natl. Acad. Sci. U.S.A.* 94 6228–6231. 10.1073/pnas.94.12.6228 9177199 PMC21031

[B42] Howard J. (ed.) (2001). *Mechanics of Motor Proteins and the Cytoskeleton.* Sunderland, MA: Sinauer Associates, Inc.

[B43] Ierushalmi N. Malik-Garbi M. Manhart A. Abu-Shah E. Goode B. L. Mogilner A. (2019). Centering and symmetry breaking in confined contracting actomyosin networks. *arXiv* [Preprint] arXiv:1907.10642. 10.7554/eLife.55368 PMC7173961 32314730

[B44] Ishihara K. Nguyen P. A. Groen A. C. Field C. M. Mitchison T. J. (2014). Microtubule nucleation remote from centrosomes may explain how asters span large cells. *Proc. Natl. Acad. Sci. U.S.A.* 111 17715–17722. 10.1073/pnas.1418796111 25468969 PMC4273342

[B45] Jegou A. Carlier M. F. Romet-Lemonne G. (2013). Formin mDia1 senses and generates mechanical forces on actin filaments. *Nat. Commun.* 4:1883. 10.1038/ncomms2888 23695677

[B46] Kimura A. Onami S. (2005). Computer simulations and image processing reveal length-dependent pulling force as the primary mechanism for *C. elegans* male pronuclear migration. *Dev. Cell* 8 765–775. 10.1016/j.devcel.2005.03.007 15866166

[B47] Kimura K. Kimura A. (2011). Intracellular organelles mediate cytoplasmic pulling force for centrosome centration in the *Caenorhabditis elegans* early embryo. *Proc. Natl. Acad. Sci. U.S.A.* 108 137–142. 10.1073/pnas.1013275108 21173218 PMC3017145

[B48] Laan L. Husson J. Munteanu E. L. Kerssemakers J. W. Dogterom M. (2008). Force-generation and dynamic instability of microtubule bundles. *Proc. Natl. Acad. Sci. U.S.A.* 105 8920–8925. 10.1073/pnas.0710311105 18577596 PMC2449340

[B49] Laan L. Pavin N. Husson J. Romet-Lemonne G. Van duijn M. López Magdalena P. (2012). Cortical dynein controls microtubule dynamics to generate pulling forces that position microtubule asters. *Cell* 148 502–514. 10.1016/j.cell.2012.01.007 22304918 PMC3292199

[B50] Laurent V. Loisel T. P. Harbeck B. Wehman A. Grobe L. Jockusch B. M. (1999). Role of proteins of the Ena/VASP family in actin-based motility of Listeria monocytogenes. *J. Cell Biol.* 144 1245–1258. 10.1083/jcb.144.6.1245 10087267 PMC2150578

[B51] Letort G. Nedelec F. Blanchoin L. Thery M. (2016). Centrosome centering and decentering by microtubule network rearrangement. *Mol. Biol. Cell* 27 2833–2843. 10.1091/mbc.E16-06-0395 27440925 PMC5025270

[B52] Li H. Guo F. Rubinstein B. Li R. (2008). Actin-driven chromosomal motility leads to symmetry breaking in mammalian meiotic oocytes. *Nat. Cell Biol.* 10 1301–1308. 10.1038/ncb1788 18836438

[B53] Liu Y. J. Le Berre M. Lautenschlaeger F. Maiuri P. Callan-Jones A. Heuze M. (2015). Confinement and low adhesion induce fast amoeboid migration of slow mesenchymal cells. *Cell* 160 659–672. 10.1016/j.cell.2015.01.007 25679760

[B54] Loisel T. P. Boujemaa R. Pantaloni D. Carlier M. F. (1999). Reconstitution of actin-based motility of *Listeria* and *Shigella* using pure proteins. *Nature* 401 613–616. 10.1038/44183 10524632

[B55] Maiuri P. Rupprecht J. F. Wieser S. Ruprecht V. Benichou O. Carpi N. (2015). Actin flows mediate a universal coupling between cell speed and cell persistence. *Cell* 161 374–386. 10.1016/j.cell.2015.01.056 25799384

[B56] Malik-Garbi M. Ierushalmi N. Jansen S. Abu-Shah E. Goode B. L. Mogilner A. (2019). Scaling behaviour in steady-state contracting actomyosin networks. *Nat. Phys.* 15 509–516. 10.1038/s41567-018-0413-4 31754369 PMC6871652

[B57] Mallik R. Carter B. C. Lex S. A. King S. J. Gross S. P. (2004). Cytoplasmic dynein functions as a gear in response to load. *Nature* 427 649–652. 10.1038/nature02293 14961123

[B58] Marcy Y. Prost J. Carlier M.-F. Sykes C. (2004). Forces generated during actin-based propulsion: a direct measurement by micromanipulation. *Proc. Nat. Acad. Sci. U.S.A.* 101 5992–5997. 10.1073/pnas.0307704101 15079054 PMC395911

[B59] Mayer M. Depken M. Bois J. S. Julicher F. Grill S. W. (2010). Anisotropies in cortical tension reveal the physical basis of polarizing cortical flows. *Nature* 467 617–621. 10.1038/nature09376 20852613

[B60] McGrath J. L. Eungdamrong N. J. Fisher C. I. Peng F. Mahadevan L. Mitchison T. J. (2003). The force-velocity relationship for the actin-based motility of *Listeria monocytogenes*. *Curr. Biol.* 13 329–332. 10.1016/s0960-9822(03)00051-4 12593799

[B61] McNally F. J. (2013). Mechanisms of spindle positioning. *J. Cell Biol.* 200 131–140. 10.1083/jcb.201210007 23337115 PMC3549965

[B62] Minc N. Burgess D. Chang F. (2011). Influence of cell geometry on division-plane positioning. *Cell* 144 414–426. 10.1016/j.cell.2011.01.016 21295701 PMC3048034

[B63] Minc N. Piel M. (2012). Predicting division plane position and orientation. *Trends Cell Biol.* 22 193–200. 10.1016/j.tcb.2012.01.003 22321291

[B64] Mitchison T. Wuhr M. Nguyen P. Ishihara K. Groen A. Field C. M. (2012). Growth, interaction, and positioning of microtubule asters in extremely large vertebrate embryo cells. *Cytoskeleton (Hoboken)* 69 738–750. 10.1002/cm.21050 22786885 PMC3690567

[B65] Morin X. Bellaiche Y. (2011). Mitotic spindle orientation in asymmetric and symmetric cell divisions during animal development. *Dev. Cell* 21 102–119. 10.1016/j.devcel.2011.06.012 21763612

[B66] Munjal A. Lecuit T. (2014). Actomyosin networks and tissue morphogenesis. *Development* 141 1789–1793. 10.1242/dev.091645 24757001

[B67] Nazockdast E. Rahimian A. Needleman D. Shelley M. (2017). Cytoplasmic flows as signatures for the mechanics of mitotic positioning. *Mol. Biol. Cell* 28 3261–3270. 10.1091/mbc.E16-02-0108 28331070 PMC5687028

[B68] Nicklas R. B. (1983). Measurements of the force produced by the mitotic spindle in anaphase. *J. Cell Biol.* 97 542–548. 10.1083/jcb.97.2.542 6885908 PMC2112533

[B69] O’Connell C. B. Wang Y. L. (2000). Mammalian spindle orientation and position respond to changes in cell shape in a dynein-dependent fashion. *Mol. Biol. Cell* 11 1765–1774. 10.1091/mbc.11.5.1765 10793150 PMC14882

[B70] Pantaloni D. Le Clainche C. Carlier M. F. (2001). Mechanism of actin-based motility. *Science* 292 1502–1506. 10.1126/science.1059975 11379633

[B71] Parekh S. H. Chaudhuri O. Theriot J. A. Fletcher D. A. (2005). Loading history determines the velocity of actin-network growth. *Nat. Cell Biol.* 7 1219–1223. 10.1038/ncb1336 16299496

[B72] Pierre A. Salle J. Wuhr M. Minc N. (2016). Generic theoretical models to predict division patterns of cleaving embryos. *Dev. Cell* 39 667–682. 10.1016/j.devcel.2016.11.018 27997824 PMC5180451

[B73] Pinot M. Steiner V. Dehapiot B. Yoo B. K. Chesnel F. Blanchoin L. (2012). Confinement induces actin flow in a meiotic cytoplasm. *Proc. Natl. Acad. Sci. U.S.A.* 109 11705–11710. 10.1073/pnas.1121583109 22753521 PMC3406835

[B74] Pizarro-Cerda J. Cossart P. (2006). Subversion of cellular functions by *Listeria monocytogenes*. *J. Pathol.* 208 215–223. 10.1002/path.1888 16362984

[B75] Placais P. Y. Balland M. Guerin T. Joanny J. F. Martin P. (2009). Spontaneous oscillations of a minimal actomyosin system under elastic loading. *Phys. Rev. Lett.* 103:158102. 19905668 10.1103/PhysRevLett.103.158102

[B76] Plastino J. Sykes C. (2005). The actin slingshot. *Curr. Opin. Cell Biol.* 17 62–66. 10.1016/j.ceb.2004.12.001 15661520

[B77] Portnoy D. A. Auerbuch V. Glomski I. J. (2002). The cell biology of *Listeria monocytogenes* infection: the intersection of bacterial pathogenesis and cell-mediated immunity. *J. Cell Biol.* 158 409–414. 12163465 10.1083/jcb.200205009 PMC2173830

[B78] Prost J. Joanny J.-F. Lenz P. Sykes C. (2008). “The physics of listeria propulsion,” in *Cell Motility*, ed. Lenz P. (New York, NY: Springer), 1–30. 10.1007/978-0-387-73050-9_1

[B79] Rai A. K. Rai A. Ramaiya A. J. Jha R. Mallik R. (2013). Molecular adaptations allow dynein to generate large collective forces inside cells. *Cell* 152 172–182. 10.1016/j.cell.2012.11.044 23332753

[B80] Rappaport R. (1967). Cell division: direct measurement of maximum tension exerted by furrow of echinoderm eggs. *Science* 156 1241–1243. 10.1126/science.156.3779.1241 6067406

[B81] Reinsch S. Gönczy P. (1998). Mechanisms of nuclear positioning. *J. Cell Sci.* 111 2283–2295. 9683624 10.1242/jcs.111.16.2283

[B82] Reymann A. C. Boujemaa-Paterski R. Martiel J. L. Guerin C. Cao W. Chin H. F. (2012). Actin network architecture can determine myosin motor activity. *Science* 336 1310–1314. 10.1126/science.1221708 22679097 PMC3649007

[B83] Ruprecht V. Wieser S. Callan-Jones A. Smutny M. Morita H. Sako K. (2015). Cortical contractility triggers a stochastic switch to fast amoeboid cell motility. *Cell* 160 673–685. 10.1016/j.cell.2015.01.008 25679761 PMC4328143

[B84] Salle J. Xie J. Ershov D. Lacassin M. Dmitrieff S. Minc N. (2019). Asymmetric division through a reduction of microtubule centering forces. *J. Cell Biol.* 218 771–782. 10.1083/jcb.201807102 30563876 PMC6400563

[B85] Sanchez T. Chen D. T. Decamp S. J. Heymann M. Dogic Z. (2012). Spontaneous motion in hierarchically assembled active matter. *Nature* 491 431–434. 10.1038/nature11591 23135402 PMC3499644

[B86] Schaller V. Weber C. Semmrich C. Frey E. Bausch A. R. (2010). Polar patterns of driven filaments. *Nature* 467 73–77. 10.1038/nature09312 20811454

[B87] Schuh M. Ellenberg J. (2008). A new model for asymmetric spindle positioning in mouse oocytes. *Curr. Biol.* 18 1986–1992. 10.1016/j.cub.2008.11.022 19062278

[B88] Sechi A. S. Wehland J. Small J. V. (1997). The isolated comet tail pseudopodium of *Listeria monocytogenes*: a tail of two actin filament populations, long and axial and short and random. *J. Cell Biol.* 137 155–167. 10.1083/jcb.137.1.155 9105044 PMC2139863

[B89] Shamipour S. Kardos R. Xue S.-L. Hof B. Hannezo E. Heisenberg C.-P. (2019). Bulk actin dynamics drive phase segregation in zebrafish oocytes. *Cell* 177 1463.e18–1479.e18. 10.1016/j.cell.2019.04.030 31080065

[B90] Shinar T. Mana M. Piano F. Shelley M. J. (2011). A model of cytoplasmically driven microtubule-based motion in the single-celled *Caenorhabditis elegans* embryo. *Proc. Nat. Acad. Sci. U.S.A.* 108 10508–10513. 10.1073/pnas.1017369108 21670261 PMC3127902

[B91] Svitkina T. (2018). The actin cytoskeleton and actin-based motility. *Cold Spring Harb. Perspect. Biol.* 10:a018267. 10.1101/cshperspect.a018267 29295889 PMC5749151

[B92] Tang S. K. Y. Renz M. Shemesh T. Driscoll M. Lippincott-Schwartz J. (2018). Cytoplasmic self-organization established by internal lipid membranes in the interplay with either actin or microtubules. *bioRxiv* [Preprint]. 10.1101/506436

[B93] Tanimoto H. Kimura A. Minc N. (2016). Shape-motion relationships of centering microtubule asters. *J. Cell Biol.* 212 777–787. 10.1083/jcb.201510064 27022090 PMC4810306

[B94] Tanimoto H. Sallé J. Dodin L. Minc N. (2018). Physical forces determining the persistency and centring precision of microtubule asters. *Nat. Phys.* 14 848–854. 10.1038/s41567-018-0154-4 30079097 PMC6071857

[B95] Taunton J. Rowning B. A. Coughlin M. L. Wu M. Moon R. T. Mitchison T. J. (2000). Actin-dependent propulsion of endosomes and lysosomes by recruitment of N-WASP. *J. Cell Biol.* 148 519–530. 10.1083/jcb.148.3.519 10662777 PMC2174808

[B96] Telley I. A. Gáspár I. Ephrussi A. Surrey T. (2012). Aster migration determines the length scale of nuclear separation in the *Drosophila* syncytial embryo. *J. Cell Biol.* 197 887–895. 10.1083/jcb.201204019 22711698 PMC3384421

[B97] Terasaki M. Jaffe L. A. (1991). Organization of the sea urchin egg endoplasmic reticulum and its reorganization at fertilization. *J. Cell Biol.* 114 929–940. 10.1083/jcb.114.5.929 1874789 PMC2289104

[B98] Theriot J. A. Mitchison T. J. Tilney L. G. Portnoy D. A. (1992). The rate of actin-based motility of intracellular *Listeria monocytogenes* equals the rate of actin polymerization. *Nature* 357 257–260. 10.1038/357257a0 1589024

[B99] Theriot J. A. Rosenblatt J. Portnoy D. A. Goldschmidt-Clermont P. J. Mitchison T. J. (1994). Involvement of profilin in the actin-based motility of *L. monocytogenes* in cells and in cell-free extracts. *Cell* 76 505–517. 10.1016/0092-8674(94)90114-7 8313471

[B100] Thoresen T. Lenz M. Gardel M. L. (2011). Reconstitution of contractile actomyosin bundles. *Biophys. J.* 100 2698–2705. 10.1016/j.bpj.2011.04.031 21641315 PMC3117186

[B101] Tilney L. G. Derosier D. J. Tilney M. S. (1992). How *Listeria* exploits host cell actin to form its own cytoskeleton. I. Formation of a tail and how that tail might be involved in movement. *J. Cell Biol.* 118 71–81. 10.1083/jcb.118.1.71 1618908 PMC2289525

[B102] Tilney L. G. Portnoy D. A. (1989). Actin filaments and the growth, movement, and spread of the intracellular bacterial parasite, *Listeria monocytogenes*. *J. Cell Biol.* 109 1597–1608. 10.1083/jcb.109.4.1597 2507553 PMC2115783

[B103] Tolić-Nørrelykke I. M. (2008). Push-me-pull-you: how microtubules organize the cell interior. *Eur. Biophys. J.* 37 1271–1278. 10.1007/s00249-008-0321-0 18404264 PMC2518947

[B104] Tolić-Nørrelykke I. M. Sacconi L. Thon G. Pavone F. S. (2004). Positioning and elongation of the fission yeast spindle by microtubule-based pushing. *Curr. Biol.* 14 1181–1186. 10.1016/j.cub.2004.06.029 15242615

[B105] Tran P. T. Marsh L. Doye V. Inoué S. Chang F. (2001). A mechanism for nuclear positioning in fission yeast based on microtubule pushing. *J. Cell Biol.* 153 397–412. 10.1083/jcb.153.2.397 11309419 PMC2169469

[B106] Trichet L. Campàs O. Sykes C. Plastino J. (2007). VASP governs actin dynamics by modulating filament anchoring. *Biophys. J.* 92 1081–1089. 10.1529/biophysj.106.091884 17098798 PMC1779978

[B107] Upadhyaya A. Chabot J. R. Andreeva A. Samadani A. Van Oudenaarden A. (2003). Probing polymerization forces by using actin-propelled lipid vesicles. *Proc. Nat. Acad. Sci. U.S.A.* 100 4521–4526. 10.1073/pnas.0837027100 12657740 PMC153588

[B108] Vallee R. B. Stehman S. A. (2005). How dynein helps the cell find its center: a servomechanical model. *Trends Cell Biol.* 15 288–294. 10.1016/j.tcb.2005.04.005 15953546

[B109] Welch M. D. Iwamatsu A. Mitchison T. J. (1997). Actin polymerization is induced by Arp2/3 protein complex at the surface of *Listeria monocytogenes*. *Nature* 385 265–269. 10.1038/385265a0 9000076

[B110] Welch M. D. Way M. (2013). Arp2/3-mediated actin-based motility: a tail of pathogen abuse. *Cell Host Microbe* 14 242–255. 10.1016/j.chom.2013.08.011 24034611 PMC3933244

[B111] Wu H.-Y. Nazockdast E. Shelley M. J. Needleman D. J. (2016). Forces positioning the mitotic spindle: theories, and now experiments. *BioEssays* 39:1600212. 10.1002/bies.201600212 28026040

[B112] Wu J. Dickinson R. B. Lele T. P. (2012). Investigation of in vivo microtubule and stress fiber mechanics with laser ablation. *Integr. Biol. (Camb)* 4 471–479. 10.1039/c2ib20015e 22495508

[B113] Wühr M. Dumont S. Groen A. C. Needleman D. J. Mitchison T. J. (2009). How does a millimeter-sized cell find its center? *Cell Cycle* 8 1115–1121. 10.4161/cc.8.8.8150 19282671 PMC2880816

[B114] Wuhr M. Tan E. S. Parker S. K. Detrich H. W. III Mitchison T. J. (2010). A model for cleavage plane determination in early amphibian and fish embryos. *Curr. Biol.* 20 2040–2045. 10.1016/j.cub.2010.10.024 21055946 PMC3031131

